# Is a single dose of commonly used antibiotics effective in preventing maternal infection after cesarean section? A network meta-analysis

**DOI:** 10.1371/journal.pone.0264438

**Published:** 2022-04-06

**Authors:** Ye Huang, Xinbo Yin, Xiaokai Wang, Fangyi Zhou, Xiaoxia Cao, Yeqiong Han, Shichang Sun

**Affiliations:** 1 School of Public Health, Shanxi Medical University, Taiyuan, Shanxi, China; 2 Department of Emergency Medicine, National Clinical Research Center of Geriatric Disorders (Xiangya Hospital), Central South University, Changsha, Hunan, China; The University of Mississippi Medical Center, UNITED STATES

## Abstract

**Objective:**

This study aimed to compare the efficacy of different antibiotic classes and dosages in preventing maternal infection after cesarean delivery.

**Methods:**

Databases were searched for randomized controlled trials (RCTs) published between January 1980 and January 2021 on antibiotic use for the prevention of maternal infection after cesarean delivery. The outcomes were endometritis, febrile morbidity, and wound infection, reported as odds ratios (OR) and surface under the cumulative ranking curve analysis scores.

**Results:**

A total of 31 RCTs met the inclusion criteria. In the network meta-analysis (NMA) for endometritis, pooled network OR values indicated that the following interventions were superior to placebo: cephalosporins (OR: 0.18, 95% credibility interval [CrI]: 0.07–0.45), penicillins (OR: 0.19, 95% CrI: 0.07–0.50), penicillins (multiple doses) (OR: 0.20, 95% CrI: 0.05–0.65), combination therapies (OR: 0.22, 95% CrI: 0.09–0.54), and cephalosporins (multiple doses) (OR: 0.25, 95% CrI: 0.08–0.74). In the NMA for febrile morbidity, placebo was more effective than the other interventions. In the NMA for wound infection, pooled network OR values indicated that the following interventions were superior to placebo: penicillin (OR: 0.14, 95% CrI: 0.05–0.37), cephalosporins (OR: 0.19, 95% CrI: 0.08–0.41), cephalosporins (multiple doses) (OR: 0.20, 95% CrI: 0.06–0.58), combination therapies (OR: 0.29, 95% CrI: 0.13–0.57), macrolides (OR: 0.33, 95% CrI: 0.15–0.74), and penicillins (multiple doses) (OR: 0.40, 95% CrI: 0.17–0.91).

**Conclusions:**

Compared with placebo, a single dose of commonly used antibiotics may prevent maternal infection after cesarean delivery. However, the incidence of febrile morbidity was not reduced.

## Introduction

Cesarean delivery is performed for many indications, including maternal desire, elective, and surgical life-saving procedures for mothers and babies in dystocia or other emergencies [1]. The World Health Organization recommends a cesarean delivery rate of ≤15% for optimal maternal and perinatal outcomes as well as due to the associated risks. However, this rate remains high in many countries [2, 3]. One of the common complications in cesarean delivery procedures is surgical site infection (SSI). Previous studies conducted between 2007 and 2011 on approximately 9,000 cesarean deliveries performed in different populations estimated that 3–12% of all cesarean deliveries were associated with SSI [4].

Prophylactic antibiotics have been associated with a 60–70% reduction in maternal infection among women who have undergone cesarean delivery [5]. The American College of Obstetricians and Gynecologists (ACOG; 2018), the Infectious Diseases Society of America (2013), and the Canadian Society of Obstetrics and Gynecology (2017) have recommended the use of first-generation cephalosporins as the first choice for prophylaxis during cesarean delivery [6]. Moreover, the Royal College of Obstetricians and Gynecologists in the United Kingdom has recommended co-amoxiclav (amoxicillin plus clavulanic acid) [6]. Although previous studies have suggested a similar efficacy across different antibiotic dosages [7, 8], there is limited evidence regarding the comparison of the efficacy of different antibiotic classes and dosages. An existing review that used meta-analysis to analyze studies suggested that cephalosporins and penicillins may have similar efficacy in preventing maternal infection after cesarean delivery [6, 7, 9]. However, this systematic review was published several years ago; hence, an update may be required.

Moreover, we aim to perform a network meta-analysis (NMA) to further examine this problem. A meta-analysis can only compare two different classes or doses of antibiotics at one time; on the other hand, an NMA can compare multiple classes and doses at once. We intend to update the literature and include studies comparing multiple antibiotic classes.

### Objective

This study aimed to employ an NMA to determine whether a single dose of commonly used antibiotics can effectively prevent maternal infection after cesarean section.

## Materials and methods

### Protocol and registration

This NMA followed the guidelines outlined in the Preferred Reporting Items for Systematic Review and Meta-Analyses (PRISMA) report [3]. The protocol used in this study was registered in the International Prospective Register of Systematic Reviews (registration number: CRD442020219455; date: 2020-12-09).

### Retrieval strategy

The retrieval strategy followed the PICO (Population, Intervention, Comparator, Outcome) format [3]: the “population” was women undergoing cesarean delivery; the “intervention” was antibiotic used to prevent maternal infection after cesarean delivery; the “comparator” was another antibiotic used to prevent maternal infection after cesarean delivery or placebo; and the “outcomes” were endometritis, febrile morbidity, and wound infection. Two authors (Y.H. and XB.Y.) independently searched the Cochrane Central Database, PubMed, Web of Science, ClinicalTrials.gov, MEDLINE, and EMBASE databases for randomized controlled trials (RCTs) on antibiotic use in the prevention of maternal infection after cesarean delivery published between January 1, 1980, and January 1, 2021. A third author (XK.W.) was consulted to resolve differences through discussion, as appropriate.

We used the following search terms:

"antibiotic prophylaxis" OR "anti-infective agents" OR "cephalosporins" OR "macrolides" OR "fluoroquinolones" OR "penicillin" OR "tetracyclines" OR "aminoglycosides" OR ("antibiotic" OR "antimicrobe" OR "anti-bacteria" OR "anti-infect") OR (["prevent" OR "prophylaxis"] AND ["bacteria" OR "infect"]) OR "placebo"
"cesarean delivery "OR "abdominal delivery" OR "surgical delivery"
"Randomized controlled trial" OR "RCT"
1) AND 2) AND 3)

### Inclusion criteria

#### Study type

Randomized controlled trial (RCT)

#### Study subjects

Women undergoing cesarean delivery, regardless of race, age, weight, etc.

### Intervention measures

1. Penicillins
2. Cephalosporins
3. Fluoroquinolones
4. Macrolides
5. Other beta-lactams (carbapenems)
6. Combination therapies
7. Placebo

### Outcome measurement

1. Endometritis
2. Febrile morbidity
3. Wound infection

### Exclusion criteria

1. Nonrandomized or pseudo-RCTs
2. Incomplete or repeated data
3. Case studies
4. Non-human studies
5. Reviews and meta-analyses

### Study selection

According to the inclusion and exclusion criteria, two authors (Y.H. and XB.Y.) independently identified potential studies from among the references of the studies yielded by the search strategy. A third author (XK.W.) was consulted to resolve differences through discussion, as appropriate.

### Data extraction

Two authors (Y.H. and XB.Y.) independently extracted relevant data using RevMan version 5.3 (Review Manager 5; https://training.cochrane.org). In case of disagreements, the original text was re-checked again and discussed to come to an agreement. If no agreement was reached, the third author (XK.W.) was consulted for arbitration. We extracted the following data parameters: name of the first author, number of patients, number of participants in each group, types of antibiotics used, dose frequency, and type of results (endometritis, febrile morbidity incidence rate, and wound infection). Moreover, the results were obtained for each arm.

### Risk of bias assessment

Two authors (Y.H. and XB.Y.) independently assessed the risk and bias for each study using RevMan version 5.3 (Review Manager 5; https://training.cochrane.org). The Cochrane Collaboration tool was used to evaluate the study quality based on the following six factors: sequence generation, allocation consideration, blind method, incomplete data, non-selective reporting of results, and other sources. Disagreements were resolved by consulting the third author (XK.W.).

### Publication bias

STATA version SE15.0 (Stata Corporation, College Station, TX, USA) was used to analyze the publication bias of the included studies by assessing comparison-adjusted funnel plots. Upon evaluating funnel plots, symmetrical distribution of the dots (representing the study) on both sides of the red line indicates a lack of publication bias and small sample effect [10].

### Outcomes

The primary outcome was endometritis, which was defined as the proportion of women with endometritis among all women undergoing cesarean delivery. It was analyzed as a binary outcome (successful or failed intervention) and reported using the network odds ratio (OR) and related 95% credibility interval (CrI). The network OR was calculated by dividing the probability of successful treatment with one class or different doses of antibiotics by the probability of successful treatment with placebo or another antibiotic class. Consequently, treatment success was defined as a network OR (including the relevant 95% CrI) of 1.0 (unified).

The secondary outcome was febrile morbidity which was defined as the number of cases of postoperative fever with various causes. The network OR was calculated by dividing the probability of successful treatment with one antibiotic class by the probability of successful treatment with placebo or another antibiotic class. Therefore, treatment success was defined as a network OR (including the relevant 95% CrI) of 1.0 (unified).

The third outcome was wound infection which was defined as the number of wound infections with various causes after cesarean delivery. The network OR was calculated by dividing the probability of successful treatment with one antibiotic class by the probability of successful treatment with placebo or another antibiotic class. Therefore, treatment success was defined as a network OR (including the relevant 95% CrI) of 1.0 (unified).

### Statistical analyses

STATA version SE15.0 (Stata Corporation, College Station, TX, USA) was first used to draw a network diagram. Subsequently, the relationship between antibiotic class and dosage was determined. Afterwards, heterogeneity analysis was conducted using R software version 3.6.1(R Project for Statistical Computing, https://www.r-project.org/). According to the Cochrane manual, when analyzing the data using a fixed-effect model, no heterogeneity was indicated for a Q-value < degrees of freedom (d.f.), *P*-value >0.10, and an I^2^ value of 0%–40%. Heterogeneity was indicated by a Q-value >d.f., *P*-value <0.10, and I^2^ >75% [10], with data analysis using a random-effect model. However, we used a random-effect model to analyze the data reliability in this NMA, regardless of heterogeneity.

Finally, NMA was conducted using the ADDIS software version 1.16.8 (Aggregate Data Drug Information System, http://www.drugis.org), which is based on a Bayesian hierarchical model. Node-splitting analysis was used to determine the model consistency. If the *P*-value was >0.05, the consistency model was used; otherwise, the inconsistency model was used [11]. Subsequently, the potential scale reduction factor (PSRF) analysis method was used to determine model convergence. When the PSRF value was 1, the model was indicated as having approximate convergence, using the network OR and 95% CrI as the effect value [12]. When both a positive result and heterogeneity were obtained, sensitivity analysis was conducted by changing the consistency model. A lack of significant change in the results would indicate that the sensitivity is low, that the results are less affected by heterogeneity, and that the results are more reliable [10].

## Results

### Study selection

**Fig 1:**
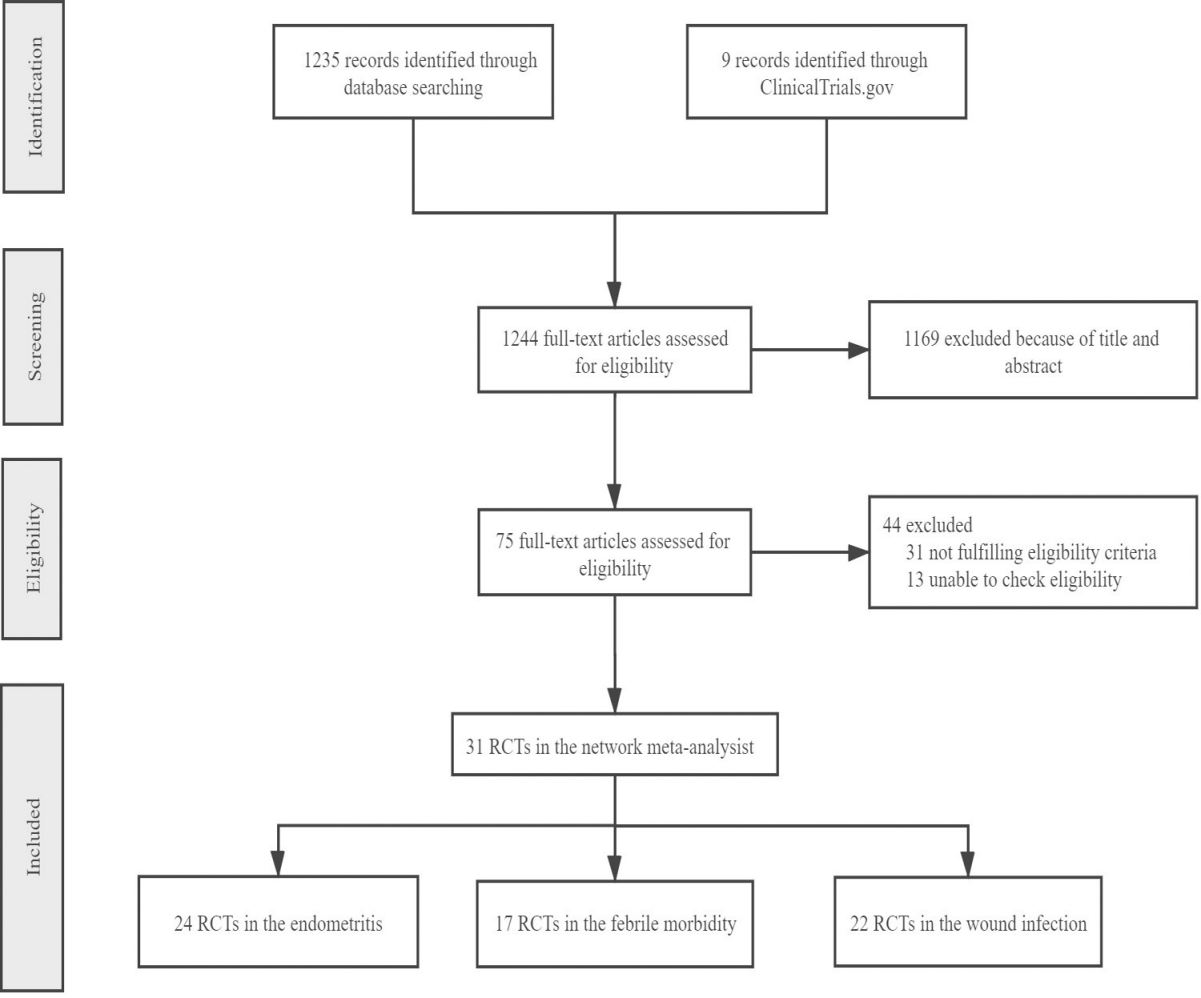
PRISMA process.

In accordance with the PRISMA standard, 1,244 RCTs were retrieved from three databases based on a search strategy. Out of these, 75 eligible studies were screened after reviewing the abstracts. After applying the inclusion and exclusion criteria, 31 RCTs were included (Fig 1).

### Characteristics of the included studies

This NMA included 31 RCTs (24 two-arm studies, 6 three-arm studies, and 1 four-arm study). The studies were published between 1980 and 2021; the majority of these were published after 1990 (Table 1). The included studies reported eight antibiotic classes and dosages, as well as placebo. With respect to the main outcome indicators, 24, 17, and 22 articles reported endometritis, febrile morbidity, and wound infection, respectively. A total of 9,707 pregnant women who underwent cesarean delivery were included. The minimum and maximum sample sizes were 48 and 2,013 cases, respectively.

**Table 1:** Characteristics of the included studies.

Study ID	Study size	Investigational drugs	Endometritis	Wound infection	Febrile morbidity
1980. Rehu	145	Placebo vs. Class F vs. Class A	√	√	
1982. Louie	181	Class B (multiple doses) vs. Class A (multiple doses)	√	√	√
1985. Saltzman	129	Class B (multiple doses) vs. Class F	√		√
1986. Beningo	283	Class B (multiple doses) vs. Class A (multiple doses)			√
1986. Dashow	204	Class B vs. Class A	√	√	√
1986. Ford	263	Class B (multiple doses) vs. Class A (multiple doses)			
1986. Saltzman	151	Class B (multiple doses) vs. Class A (multiple doses) vs. Class A	√	√	√
1988. Rosaschino	59	Class B vs. Class A		√	
1989. Mansueto	48	Class B (multiple doses) vs. Class E	√	√	√
1990. Faro	1580	Class B (multiple doses) vs. Class B vs. Class A	√		
1990. Lewis	287	Class B vs. Class A	√	√	√
1992. Koppel	119	Class B vs. Class F	√	√	
1992. Ng	220	Class B (multiple doses) vs. Placebo vs. Class A (multiple doses)		√	
1993. Chantharojwong	106	Class B (multiple doses) vs. Class A (multiple doses)	√	√	√
1994. Lumbiganon	379	Class B vs. Class F			√
1997. Bracero	170	Class B vs. Class F	√	√	√
1998. Noyes	292	Class B vs. Class F	√		
1998. Shah	184	Placebo vs. Class B (multiple doses) vs. Class F vs. Class A	√	√	√
1999. Lehapa	233	Class A (multiple doses) vs. Class B		√	
2000. Busowski	114	Class B vs. Class F vs. Class C	√	√	
2000. Spinnato	298	Cephalosporins, combination therapies, penicillin	√		
2001. Parulekar	200	Class B vs. Class F	√		√
2004. Ahmed	200	Class B vs. Class F	√	√	√
2010. Jyothi	122	Class B vs. Class F	√	√	
2010. Ziogos	176	Class B vs. Class F	√	√	
2012. Kamilya	746	Class B vs. Class F	√	√	√
2013. Mothilal	70	Class B vs. Class D			√
2014. Mivumbi	132	Class B vs. Class A	√	√	√
2016. Tita	2013	Placebo vs. Class D	√	√	
2017. Valent	403	Placebo vs. Class F	√	√	√
2020. Arshad	200	Class B vs. Class F		√	

Class A, Penicillins; Class B, Cephalosporins; Class C, Fluoroquinolones; Class D, Macrolides; Class E, Other beta-lactams (carbapenems); Class F, Combination therapies.

### Risk-of-bias and quality-of-evidence assessments

**Fig 2:**
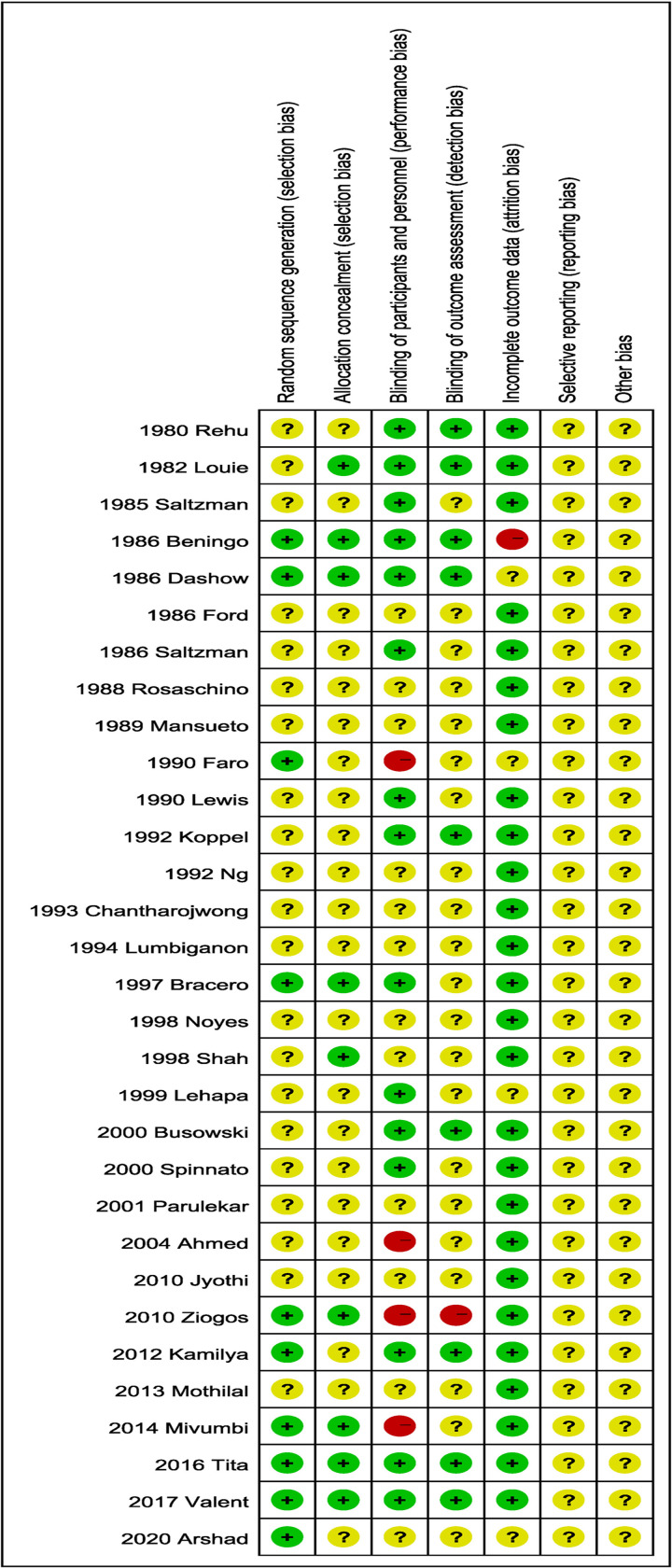
Risk-of-bias summary.

The risk-of-bias and quality-of-evidence assessments for the included studies were performed using the Cochrane bias risk assessment tool. All the included articles were RCTs. Furthermore, 84% of the studies were rated as having a low risk-of-bias, and 11 of the included RCTs described specific methods for randomization of patients and interventions. A risk-of-bias summary of the included trials is presented in Fig 2.

### Synthesis of results

#### NMA for endometritis

**Fig 3:**
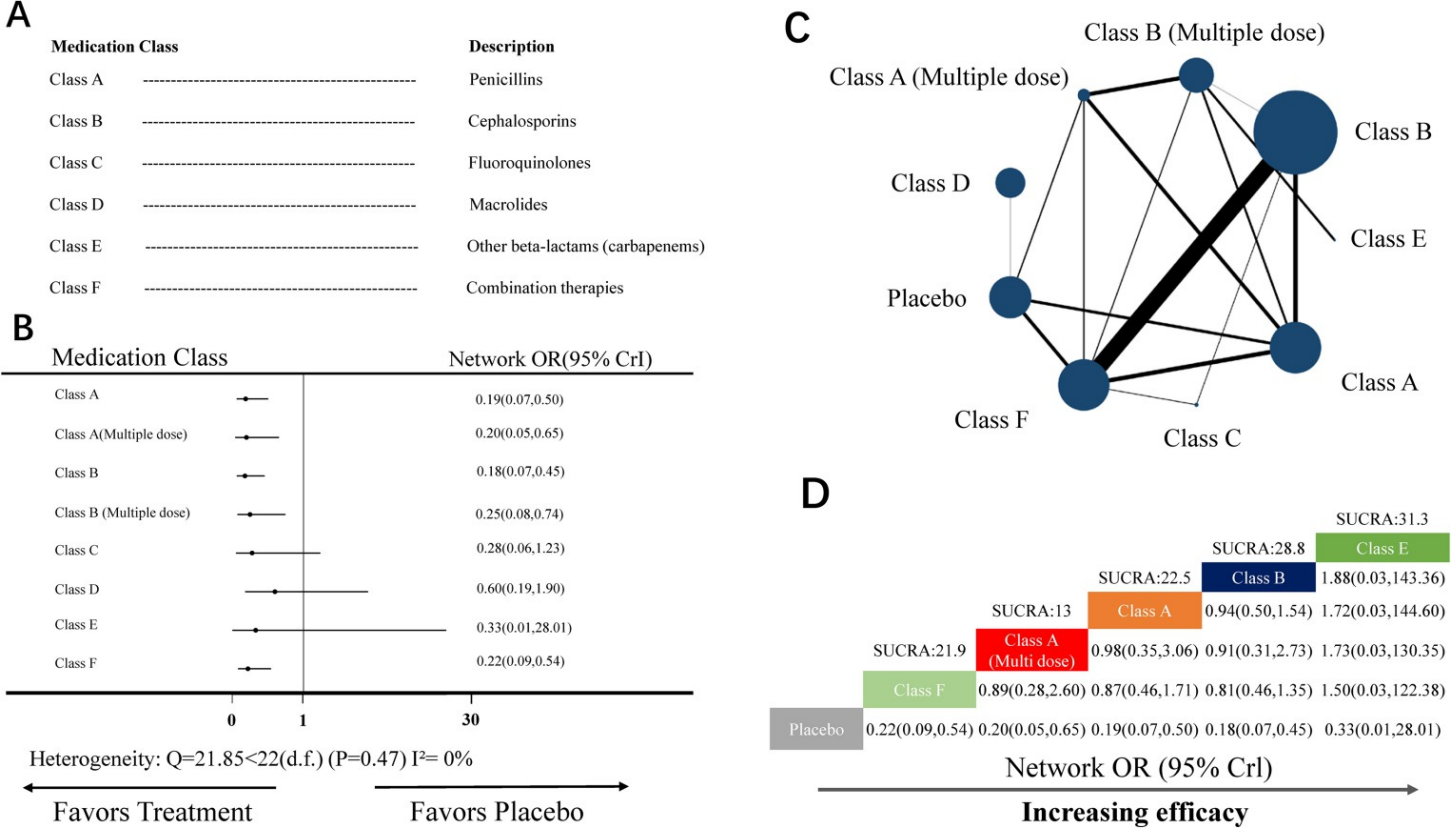
NMA for endometritis. (A) List of the included medication classes. (B) Forest plot of the NMA comparing each intervention against placebo. (C) Each node (blue circles) represents a unique medication class and dosage, and the size of each node represents the number of included women for the intervention. The connecting line indicates the number of direct comparisons between both nodes. The width of each line represents the number of direct comparisons between interventions. (D) Schematic detailing the most efficacious antibiotic class and dosage in NMA for endometritis compared with placebo for surface under the cumulative ranking curve analysis (SUCRA). NMA, network meta-analysis; OR, odds ratio; CrI, credibility interval.

The NMA for endometritis included 24 RCTs (18 two-arm studies, 5 three-arm studies, 1 four-arm study) covering eight medication classes or placebo (Fig 3A). Nine nodes were included in the NMA. Each node represented a unique medication class and doses or placebo, and the size of each node represented the included patients for the intervention (Fig 3C). Combination therapies (class F, 19 head-to-head comparisons) and cephalosporins (class B, 17 head-to-head comparisons) were the most investigated classes.

Heterogeneity analysis indicated no heterogeneity (Q-value = 21.85 <22 [d.f.], *P*-value = 0.47, I^2^ = 0%) (Fig 3B). Therefore, the random-effect model was used to analyze data.

In the NMA, the node-splitting analysis showed that both *P*-values were >0.05 (S1 Table). Therefore, we used the consistency-type model for data analysis. After 100,000 simulation iterations, the PSRF value was 1, which indicated that approximate convergence was achieved. Pooled network OR values indicated that the following interventions were superior to placebo (Fig 3B): cephalosporins (Class B; network OR: 0.18, 95% CrI: 0.07–0.45), penicillins (Class A; network OR: 0.19, 95% CrI: 0.07–0.50), penicillins (multiple doses) (Class A multi doses; network OR: 0.20, 95% CrI: 0.05–0.65), combination therapies (Class F; network OR: 0.22, 95% CrI: 0.09–0.54), and cephalosporins (multiple doses) (Class B multi doses; network OR: 0.25, 95% CrI: 0.08–0.74). Despite being equivalent to placebo, the surface under the cumulative ranking curve analysis (SUCRA) score showed that the top-ranked classes for endometritis were occupied by other beta-lactams (Class E; SUCRA score: 31.3, network OR: 0.33, 95% CrI: 0.01–28.01; Fig 3D). There was no evidence of publication bias (S1 Fig).

#### NMA for febrile morbidity

**Fig 4:**
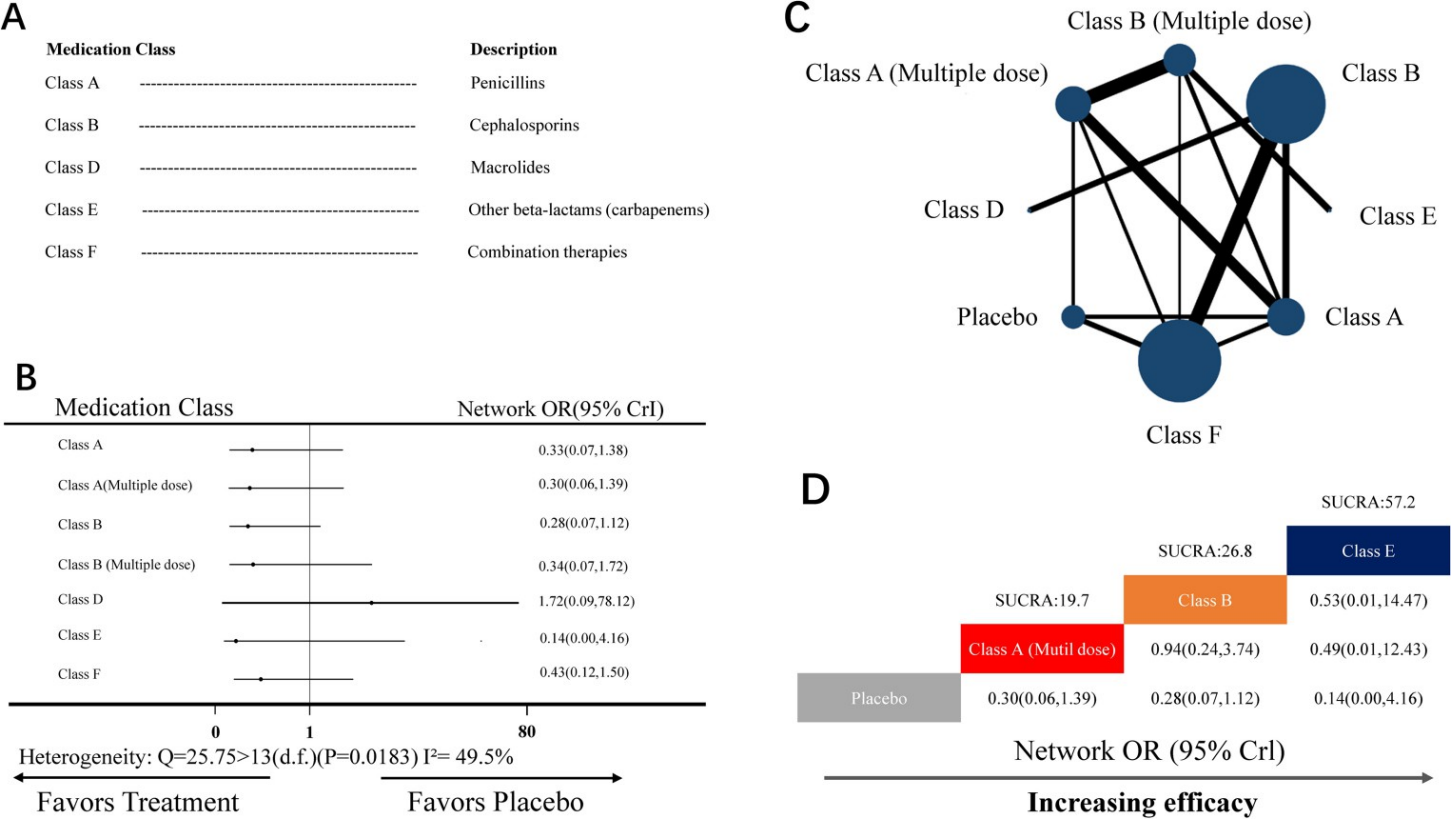
NMA for febrile morbidity. (A) List of the included medication classes. (B) Forest plot of the NMA comparing each intervention against placebo. (C) Each node (blue circles) represents a unique medication class and dosage, and the size of each node represents the number of included women for the intervention. The connecting line indicates the number of direct comparisons between both nodes. The width of each line represents the number of direct comparisons between interventions. (D) Schematic detailing the most efficacious antibiotic class and dosage in NMA for febrile morbidity compared with placebo for surface under the cumulative ranking curve analysis (SUCRA). NMA, network meta-analysis; OR, odds ratio; CrI, credibility interval.

The NMA for febrile morbidity included 17 RCTs (15 two-arm studies, 3 three-arm studies, 1 four-arm study) covering seven medication classes and dosages, as well as placebo (Fig 4A). Eight nodes were included in the NMA. Each node represented a unique medication class and doses or placebo, and the size of each node represented the included patients for the intervention (Fig 4C). Combination therapies (class F, 11 head-to-head comparisons) and cephalosporins (class B, 9 head-to-head comparisons) were the most investigated classes.

Heterogeneity analysis indicated moderate heterogeneity (Q-value = 22.75 >13 [d.f.], *P*-value = 0.0183, I^2^ = 49.5%) (Fig 4B). Therefore, the random-effect model was used to analyze data.

In the NMA, the node-splitting analysis showed that both *P*-values were >0.05 (S2 Table); therefore, the consistency-type model was used for data analysis. After 50,000 simulation iterations, the PSRF value was 1, which indicated that approximate convergence was achieved. Pooled network OR values indicated that no intervention was superior to placebo (Fig 4B). The SUCRA score revealed that the top-ranked classes for febrile morbidity were other beta-lactams (Class E; SUCRA score: 57.2, network OR: 0.14, 95% CrI: 0.00–4.16; Fig 4D). There was no evidence of publication bias (S2 Fig). Due to the lack of positive results, sensitivity analysis was not performed.

#### NMA for wound infection

**Fig 5:**
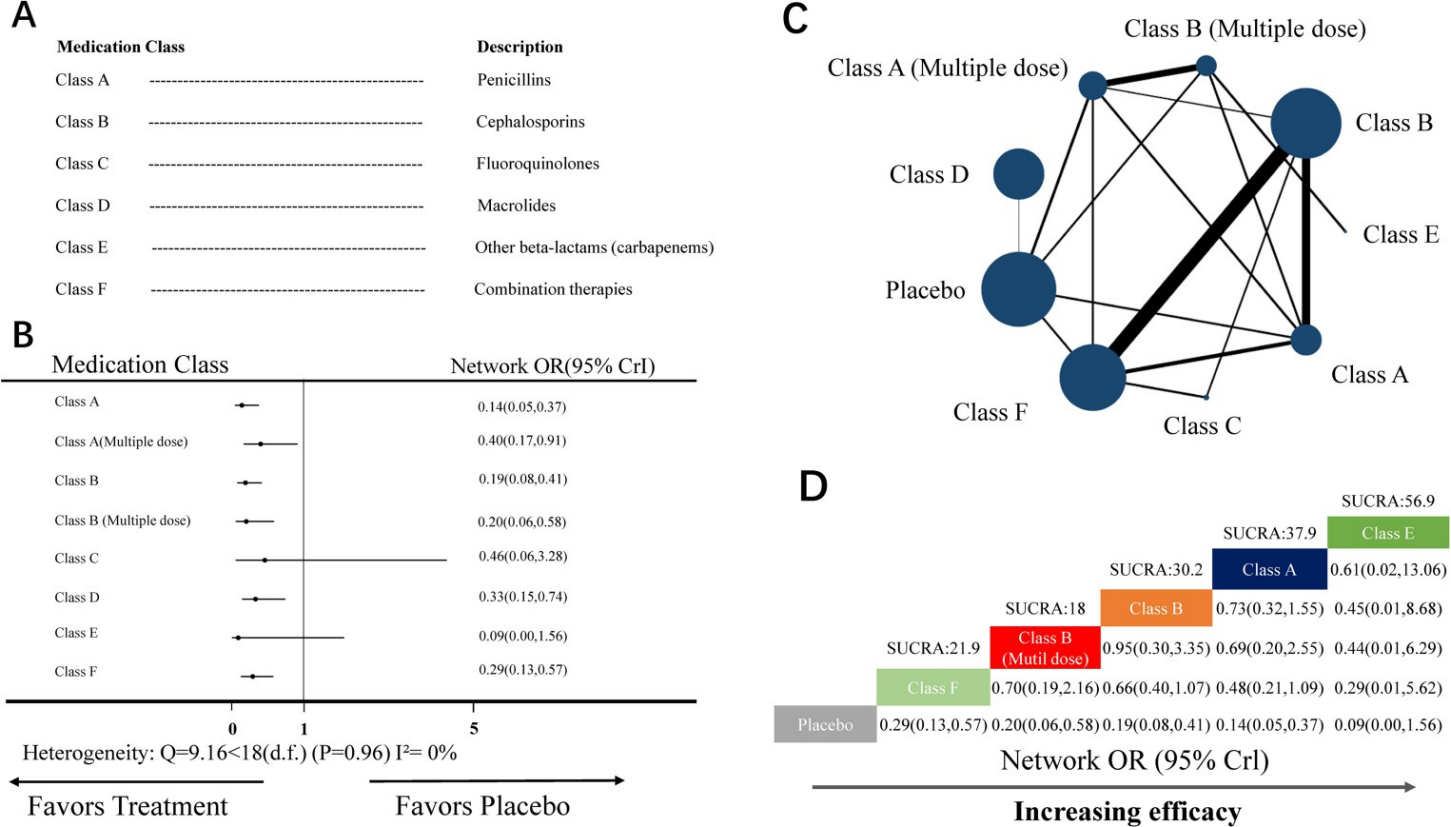
NMA for wound infection. (A) List of the included medication classes. (B) Forest plot of the NMA comparing each intervention against placebo. (C) Each node (blue circles) represents a unique medication class and dosage, and the size of each node represents the number of included women for the intervention. The connecting line indicates the number of direct comparisons between both nodes. The width of each line represents the number of direct comparisons between interventions. (D) Schematic detailing the most efficacious antibiotic class and dosage in NMA for wound infection compared with placebo for surface under the cumulative ranking curve analysis (SUCRA). NMA, network meta-analysis; OR, odds ratio; CrI, credibility interval.

The NMA for wound infection included 22 RCTs (19 two-arm studies, 2 three-arm studies, and 1 four-arm study) covering eight medication classes and placebo (Fig 5A). Nine nodes were included in the NMA. Each node represented a unique medication class and doses or placebo, and the size of each node represented the included patients for the intervention (Fig 5C). Combination therapies (class F, 15 head-to-head comparisons) and cephalosporins (class B, 14 head-to-head comparisons) were the most investigated classes.

Heterogeneity analysis indicated no heterogeneity (Q-value = 9.16 <18 [d.f.], *P*-value = 0.96, I^2^ = 0%) (Fig 5B). Therefore, the random-effect model was used for data analysis.

In the NMA, the node-splitting analysis showed that both *P*-values were >0.05 (S3 Table); therefore, we used a consistency-type model for data analysis. After 800,000 simulation iterations, the PSRF value was 1, indicating approximate convergence. Pooled network OR values showed that the following interventions were superior to placebo (Fig 5B): penicillins (Class A; network OR: 0.14, 95% CrI: 0.05–0.37), cephalosporins (Class B; network OR: 0.19, 95% CrI: 0.08–0.41), cephalosporins (multiple doses) (Class B multi doses; network OR: 0.20, 95% CrI: 0.06–0.58), combination therapies (Class F; network OR: 0.29, 95% CrI: 0.13–0.57), macrolides (Class D; network OR: 0.33, 95% CrI: 0.15–0.74), and penicillins (multiple doses) (Class A multi doses; network OR: 0.40, 95% CrI: 0.17–0.91). Despite being equivalent to placebo, the SUCRA score showed that the top-ranked classes for wound infection were other beta-lactams (Class E; SUCRA score: 56.9, network OR: 0.09, 95% CrI: 0.00–1.56; Fig 5D). There was no evidence of publication bias (S3 Fig).

## Discussion

### Principal findings

The present study was based on a total of 31 RCTs involving 9,707 women with cesarean delivery. Based on the results, cephalosporins, penicillins, penicillins (multiple doses), combination therapies, and cephalosporins (multiple doses) were identified to be superior to placebo for treating endometritis. On the other hand, no intervention was superior to placebo for febrile morbidity. Moreover, penicillins, cephalosporins, cephalosporins (multiple doses), combination therapies, macrolides, and penicillins (multiple doses) were superior to placebo to treat wound infection. Our findings suggested that a single dose of penicillin or cephalosporins can sufficiently prevent maternal infection after cesarean delivery. Assessment of SUCRA scores showed that the top-ranked antibiotic classes to treat endometritis and wound infection were other beta-lactams, which could be attributed to the small sample size and wide CrI. Therefore, the statistical indicators of clinical advantage in this study should be interpreted carefully. In the NMA for febrile morbidity, no intervention was superior to placebo. This may be related to body weight, various complications, and obstetric interventions [13], which requires further research.

Infection risk in cesarean delivery, accompanied by the use of antibiotics, is five times higher than that in vaginal delivery [14]. Numerous studies have reported that different antibiotic classes or dosages reduce the incidence of maternal infection after cesarean delivery. However, the optimal antibiotic classes and dosages remain unclear.

### Comparison with existing literature

A previous systematic review conducted by Pinto-Lopes et al. [7] reported that there is no significant difference between single and multiple antibiotic doses in terms of the prevention of maternal infection after cesarean delivery. In 2014, Smaill et al. [6] compared different antibiotic classes with placebo routinely provided to women for the prevention of infection after cesarean delivery and reported a positive result. However, this study focused only on the comparison of head-to-head antibiotic classes and was published 7 years ago.

In this study, we updated the included literature and combined numerous published RCTs in the NMA, which had a broader base. Several antibiotic classes and dosages were comprehensively evaluated, and direct and indirect comparisons were integrated. This study, which qualitatively compared different antibiotic classes and dosages, is clinically significant because choosing the appropriate antibiotics for the prevention of maternal infection after cesarean delivery is a major challenge among obstetricians [15]. Moreover, it should be noted that drug resistance caused by antibiotic overuse is a new significant problem [16–18]. Single-dose therapy can reduce the economic burden on patients and the workload of medical staff [19]. On the other hand, complex antibiotic doses increase the risk of antibiotic abuse, which results in drug resistance [7, 20, 21]. This is consistent with the ACOG Practice Bulletin recommendation [15]. Additionally, the single use of antibiotics is cheaper and more convenient for administration [8]. This further supports our conclusion that multiple doses of antibiotics are unnecessary for preventing maternal infection after cesarean delivery.

### Strengths and limitations

This study has some limitations. First, an NMA, similar to all secondary analyses, should only combine the results of similar studies. Quantifying factors that lead to non-statistical heterogeneity (e.g., drug differences within antibiotic categories, differences in research settings) is difficult; therefore, there may be some unknown bias.

Second, we did not perform subgroup analysis, since the positive results of this study did not show heterogeneity. In addition, we believe that subgroup analysis will not get positive results because recent studies comparing antibiotic use between intravenous injection and lavage groups, as well as before and after umbilical cord clamping and skin incision, have reported no significant differences between drug deliveries [9, 22, 23]. Another recent meta-analysis that investigated the use of cephalosporin generations and penicillin types to prevent maternal infection after cesarean delivery also did not find significant differences between these subgroups [6, 9].

Finally, post-delivery SSI can affect the infection rate—attributable to different hospitals at different times, leading to a bias, and this bias will be considered in our NMA.

## Conclusions

Our findings suggest that a single dose of commonly used antibiotics (including penicillin or cephalosporin) may sufficiently prevent maternal infection after cesarean delivery.

## Supporting information

S1 Checklist (DOCX)

S1 Fig Funnel plot for endometritis. (TIF)

S2 Fig Funnel plot for febrile morbidity. (TIF)

S3 Fig Funnel plot for wound infection. (TIF)

S1 Table Node-splitting analysis for endometritis. Class A, Penicillins; Class B, Cephalosporins; Class C, Fluoroquinolones; Class D, Macrolides; Class E, Other beta-lactams (carbapenems); Class F, Combination therapies.
(XLSX)

S2 Table Node-splitting analysis for febrile morbidity. Class A, Penicillins; Class B, Cephalosporins; Class C, Fluoroquinolones; Class D, Macrolides; Class E, Other beta-lactams (carbapenems); Class F, Combination therapies.
(XLSX)

S3 Table Node-splitting analysis for wound infection. Class A, Penicillins; Class B, Cephalosporins; Class C, Fluoroquinolones; Class D, Macrolides; Class E, Other beta-lactams (carbapenems); Class F, Combination therapies.
(XLSX)
