# Supplementary figures and images for: Is a single dose of commonly used antibiotics effective in preventing maternal infection after cesarean section? A network meta-analysis
Source: PLoS One. 2022 Apr 6;17(4):e0264438. doi: 10.1371/journal.pone.0264438 (PMC8985944; doi:10.1371/journal.pone.0264438)

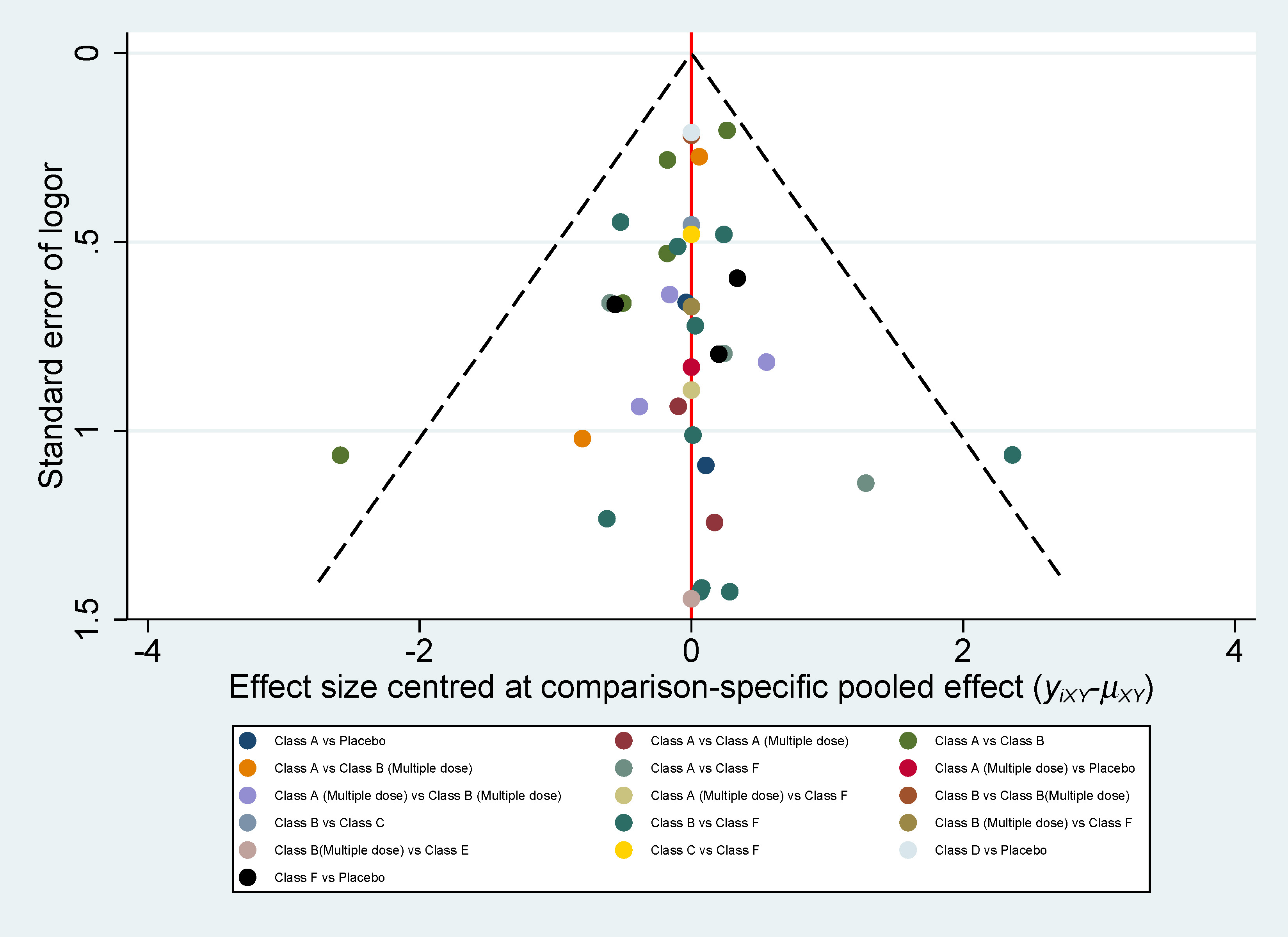

Supplement: S1 Fig — (TIF) [file pone.0264438.s002.tif]

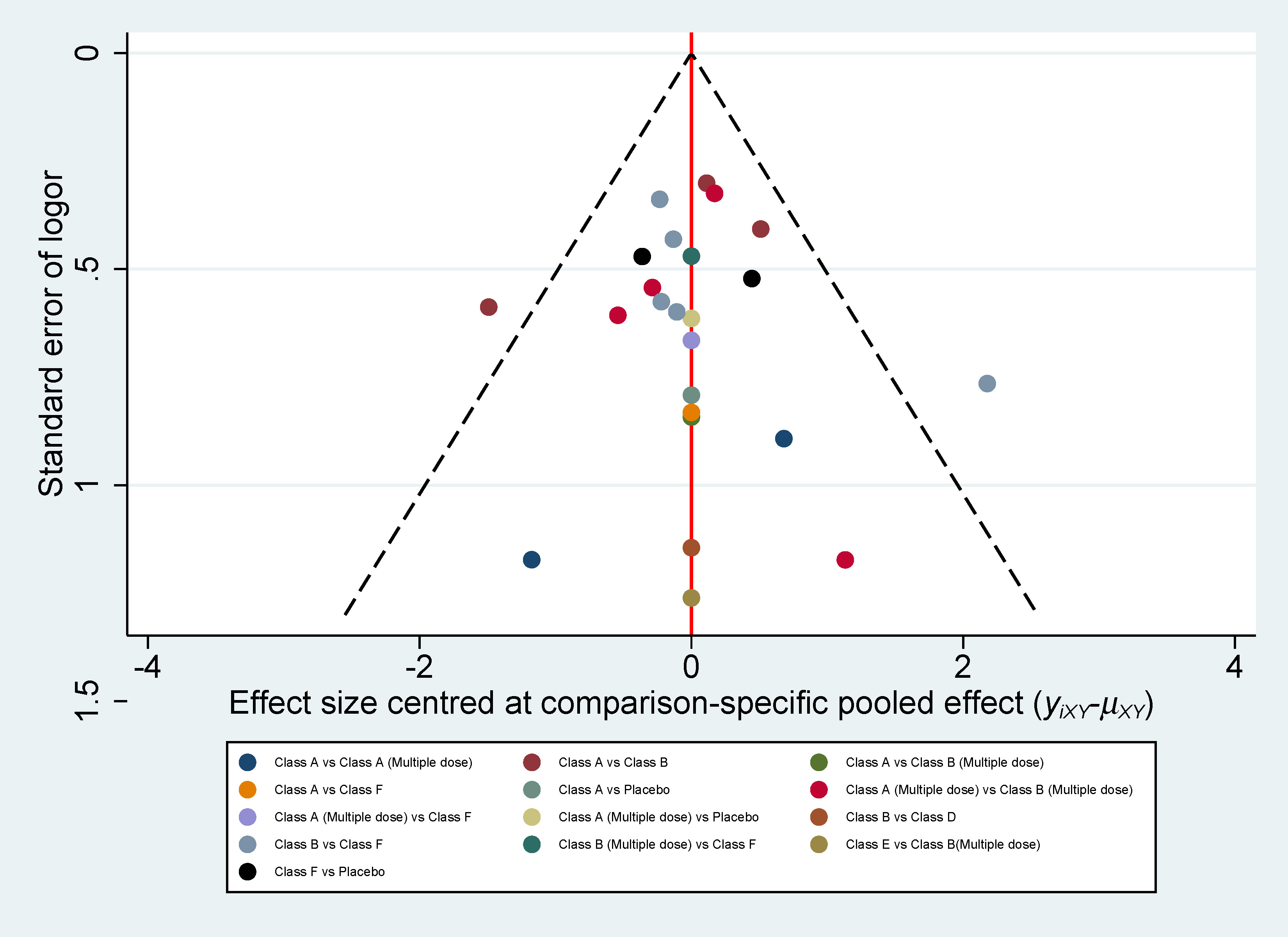

Supplement: S2 Fig — (TIF) [file pone.0264438.s003.tif]

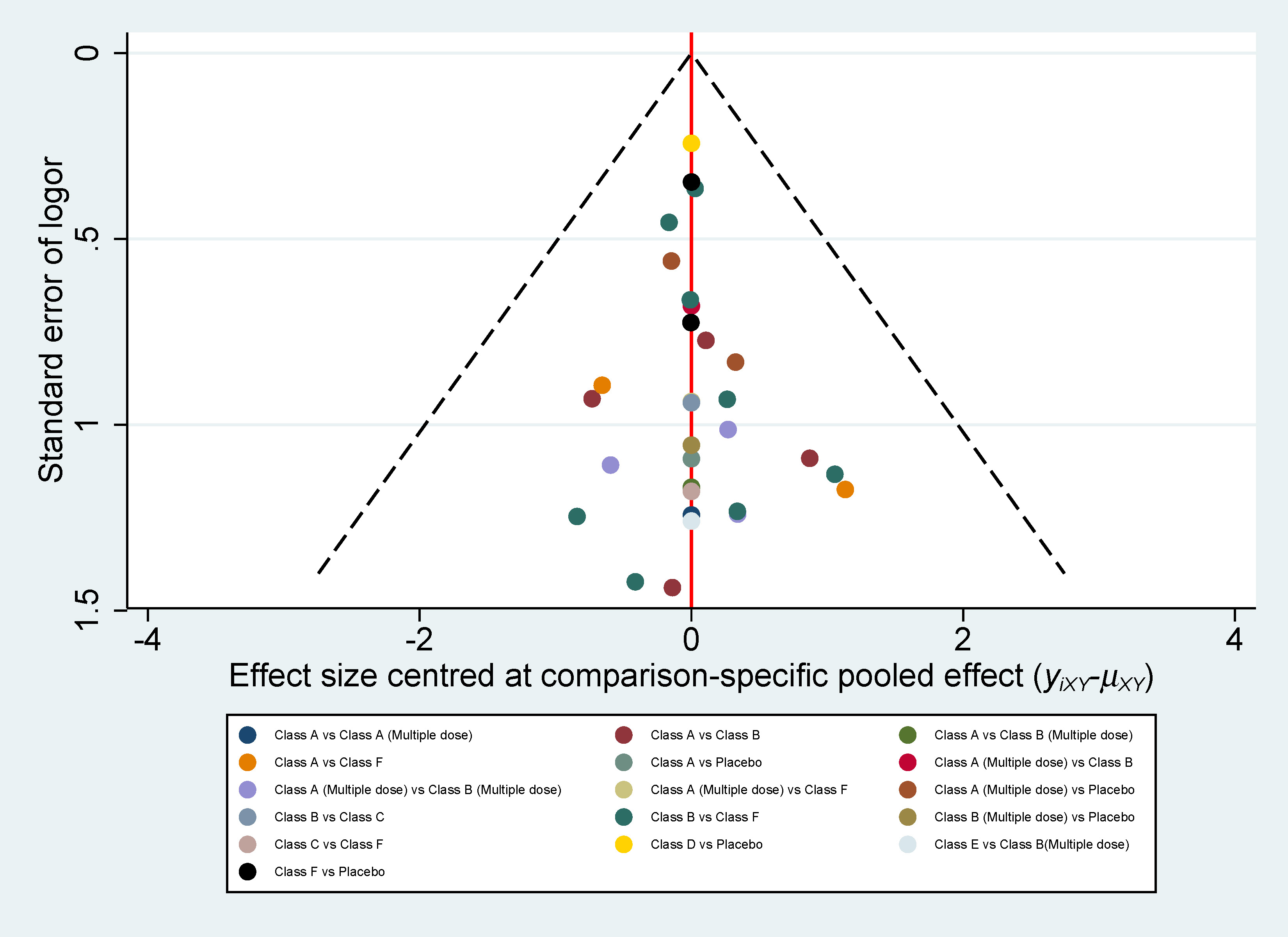

Supplement: S3 Fig — (TIF) [file pone.0264438.s004.tif]
